# Perinatal Air Pollutant Exposures and Autism Spectrum Disorder in the Children of Nurses’ Health Study II Participants

**DOI:** 10.1289/ehp.1206187

**Published:** 2013-06-18

**Authors:** Andrea L. Roberts, Kristen Lyall, Jaime E. Hart, Francine Laden, Allan C. Just, Jennifer F. Bobb, Karestan C. Koenen, Alberto Ascherio, Marc G. Weisskopf

**Affiliations:** 1 Department of Social and Behavioral Sciences, and; 2 Department of Nutrition, Harvard School of Public Health, Boston, Massachusetts, USA; 3 University of California, MIND Institute, Davis, California, USA; 4 Channing Division of Network Medicine, Brigham and Women’s Hospital and Harvard Medical School, Boston, Massachusetts, USA; 5 Department of Environmental Health,; 6 Department of Epidemiology, and; 7 Department of Biostatistics, Harvard School of Public Health, Boston, Massachusetts, USA; 8 Department of Epidemiology, Mailman School of Public Health, Columbia University, New York, New York, USA

**Keywords:** air pollution, autism, diesel, heavy metals, prenatal exposure

## Abstract

Objective: Air pollution contains many toxicants known to affect neurological function and to have effects on the fetus *in utero*. Recent studies have reported associations between perinatal exposure to air pollutants and autism spectrum disorder (ASD) in children. We tested the hypothesis that perinatal exposure to air pollutants is associated with ASD, focusing on pollutants associated with ASD in prior studies.

Methods: We estimated associations between U.S. Environmental Protection Agency–modeled levels of hazardous air pollutants at the time and place of birth and ASD in the children of participants in the Nurses’ Health Study II (325 cases, 22,101 controls). Our analyses focused on pollutants associated with ASD in prior research. We accounted for possible confounding and ascertainment bias by adjusting for family-level socioeconomic status (maternal grandparents’ education) and census tract–level socioeconomic measures (e.g., tract median income and percent college educated), as well as maternal age at birth and year of birth. We also examined possible differences in the relationship between ASD and pollutant exposures by child’s sex.

Results: Perinatal exposures to the highest versus lowest quintile of diesel, lead, manganese, mercury, methylene chloride, and an overall measure of metals were significantly associated with ASD, with odds ratios ranging from 1.5 (for overall metals measure) to 2.0 (for diesel and mercury). In addition, linear trends were positive and statistically significant for these exposures (*p* < .05 for each). For most pollutants, associations were stronger for boys (279 cases) than for girls (46 cases) and significantly different according to sex.

Conclusions: Perinatal exposure to air pollutants may increase risk for ASD. Additionally, future studies should consider sex-specific biological pathways connecting perinatal exposure to pollutants with ASD.

## Introduction

Autism spectrum disorder (ASD) is a neurodevelopmental disorder characterized by impairments in communication and social skills beginning before 3 years of age. Although ASD etiology is poorly understood, environmental exposures during gestation in particular have been implicated in the etiology of ASD (Gardener et al. 2009; Larsson et al. 2005; Roberts et al. 2007).

Air pollution contains many toxicants known to affect neurological function and to have effects on the fetus *in utero* [U.S. Environmental Protection Agency (EPA) 2010]. Several recent studies have reported associations between perinatal exposure to air pollution and ASD in children. (Kalkbrenner et al. 2010; Palmer et al. 2009; Volk et al. 2011; Windham et al. 2006). In this study, we tested the hypothesis that perinatal exposure to hazardous air pollutants increases risk of ASD by estimating associations between the U.S. EPA–modeled levels of hazardous air pollutants at the time and place of birth and ASD in the children of participants in a national prospective longitudinal cohort, the Nurses’ Health Study II, focusing our analysis on toxicants associated with ASD in prior studies.

In previous studies, metals (antimony, arsenic, cadmium, chromium, lead, mercury, manganese, nickel) (Palmer et al. 2009; Windham et al. 2006), styrene (Kalkbrenner et al. 2010), quinoline (Kalkbrenner et al. 2010), trichloroethylene (Windham et al. 2006), methylene chloride (Kalkbrenner et al. 2010; Windham et al. 2006), vinyl chloride (Windham et al. 2006), and diesel particulate matter (Volk et al. 2011; Windham et al. 2006) have been associated with ASD. U.S. EPA reviews have indicated that all of these pollutants have established or suspected effects on the nervous system and on the developing fetus from human or animal studies, except for nickel, which has no known effects on the nervous system, and quinoline, for which possible developmental effects have not been studied (U.S. EPA 2010). Arsenic, cadmium, chromium, mercury, methylene chloride, nickel, styrene, trichloroethylene, and vinyl chloride are also known or suspected mutagens (Agency for Toxic Substances and Disease Registry 2011), and *de novo* DNA mutations have been implicated in ASD etiology (Kinney et al. 2010; Sebat et al. 2007; Smith et al. 2009). Therefore, we focused on these pollutants *a priori*.

We also examined whether there are sex differences in associations between pollutants and ASD. Sex-specific etiological subtypes of ASD (Fombonne 2007) and sex differences in the association of environmental toxicants with executive function (Braun et al. 2011) have been suggested by prior research. The one previous study that reported associations between exposure to pollutants and ASD according to sex did not find statistically significant differences (Kalkbrenner et al. 2010).

## Methods

*Selection of cases and controls*. We used data from the Nurses’ Health Study II, a cohort of 116,430 female nurses from 14 U.S. states that was established in 1989 and has been followed over time with biennial questionnaires. Initially nurses were recruited from 14 U.S. states, but since that time they have moved throughout the United States. Thus, children in the current analyses were born in all 50 U.S. states. In the 2005 questionnaire, respondents were asked “Have any of your children been diagnosed with the following diseases?” with autism, Asperger’s syndrome, or other ASD listed as separate responses. In 2007–2008, we sent a questionnaire to the 756 women who had reported having a child with any of these conditions, querying the affected child’s sex, birth date, and whether they were adopted. In addition, they were asked “What ASD diagnosis has the child been given?” with autism, Asperger syndrome, and PDD-NOS (PDD not otherwise specified) as possible answers. Women were also asked about other diagnoses (such as attention deficit hyperactivity disorder and obsessive compulsive disorder) (response rate = 84%, *n* = 636). The Partners Healthcare Institutional Review Board approved this research. Completion and return of questionnaires sent by U.S. mail constituted implied consent.

Cases were excluded for the following overlapping reasons: women reported on the follow-up questionnaire that the child did not have ASD (*n* = 32); the child was adopted (*n* = 9); they did not want to participate (*n* = 20); or they did not report the child’s birth year (*n* = 71). Children reported to have trisomy 18, fragile X, an XXY genotype, or Down’s, Angelman’s, Jacobsen’s, or Rett’s syndrome also were excluded (*n* = 11). Of the remaining children, 329 were born after 1987, when air pollution data were available, but 4 had insufficient address information for geocoding, yielding 325 cases. In this study we refer to children with autism, Asperger’s syndrome, or other autism spectrum disorder as cases and use “ASD” to refer to this case definition.

We validated the ASD diagnosis by telephone administration of the Autism Diagnostic Interview–Revised (ADI-R) (Lord et al. 1994) to 50 randomly selected case mothers who indicated willingness to complete the interview (81% of the 636 mothers who responded to the follow up questionnaire were willing to be interviewed). Diagnoses reported by women who were willing versus unwilling to participate in the substudy were similar (25% autism, 51% Asperger’s, and 25% PDD-NOS compared with 25% autism, 49% Asperger’s, and 23% PDD-NOS, respectively). For the subsample of mothers who completed the ADI-R, 43 children (86%) met full ADI-R criteria for an autism diagnosis (based on minimum scores in all three domains and onset by 3 years of age). The remaining children met the onset criterion and communication domain score, but missed full diagnosis by one point in one domain (*n* = 5) or had qualifying scores in one or two domains only (*n* = 2). Thus, all of the children in the subsample exhibited some autistic behaviors and may have been on the autism spectrum.

Controls were children born during 1987–2002 (the years when air pollution data were available) to mothers who indicated that they never had a child with ASD on the 2005 questionnaire and who responded to a supplemental 2001–2002 questionnaire that queried the calendar year and sex of each of their live births and whether they smoked during pregnancy. We randomly selected one child per mother if more than one child was eligible. Of 25,828 potential controls, 3,711 were excluded because of insufficient address information, and 19 were excluded because their mothers reported that they had ASD on the 2009 questionnaire, leaving 22,098 controls. We did not include the 19 new cases because we did not follow up with mothers to confirm their case status.

*Geocoding*. The mailing address used for the biennial Nurses’ Health Study II questionnaire at the approximate time of the index child’s birth was geocoded and classified according to state, county, and census tract identifier. Children born from 1987 through 1990 were assigned the geographic location of their mother in 1989 (the first year of study). Children born in 1991 or 1992 were assigned the mother’s mailing address in 1991, and births from 1993 through 2002 were assigned the nurses’ addresses, updated every other year, in similar manner.

*Exposure assessment*. Hazardous air pollutant (HAP) concentrations were assessed by the U.S. EPA National Air Toxics Assessments in 1990, 1996, 1999, and 2002, which uses an inventory of outdoor sources of air pollution, including both stationary sources (e.g., waste incinerators, small businesses) and mobile sources (e.g., traffic) to estimate average ambient concentrations of pollutants for each census tract based on dispersion models (U.S. EPA 2011). Data were downloaded from the U.S. EPA website on 23 June 2010; additional archived data was received on compact disc from the U.S. EPA. Air pollution concentrations were linked to nurses’ residential locations at the time of the birth of their child through census tract codes (Neighborhood Change Database 2013). Children were assigned pollution concentrations from the U.S. EPA assessment closest to their year of birth (births 1987–1993 used 1990 concentrations; births 1994–1997 used 1996 concentrations; births 1998–2000 used 1999 concentrations; births 2001–2002 used 2002 concentrations). We categorized each pollutant according to quintiles of concentration in the entire study population.

*Covariates*. We examined family and community socioeconomic indicators that may be associated with ASD ascertainment. To characterize community circumstances around the time by which ASD was likely to have been diagnosed, we used two U.S. Census tract variables (linked by mother’s mailing address) measured 6 years after the birth of the child: median income and percent of residents with a college education, which we divided into quartiles. We used the maximum of the mother’s parents’ education during her infancy as a proxy measure of maternal childhood socioeconomic status. The index child’s current family income was based on the family income reported by the mother in 2001. The educational attainment of the mother’s partner or spouse was reported in 1999. We also examined factors that may be associated with both ASD and air pollutant exposure: smoking, year of birth, maternal age at birth, and air pollution prediction model year. Smoking during the index pregnancy was assessed in 2001. Year of birth was by mother’s report. Maternal age at birth was calculated by subtracting the child’s birth year from the mother’s birth year. The air pollution prediction model year (HAP year), 1990, 1996, 1999, or 2002, was modeled as a categorical variable.

*Analyses*. We examined the association of demographic covariates with ASD case status to assess possible confounding. To calculate odds ratios (ORs) for ASD associated with exposure to specific pollutants, we fit separate logistic regression models with ASD case status as the dependent variable and quintiles of each pollutant as the independent variable, both adjusted for child’s sex and stratified by sex. To test a linear dose–response relationship of pollutant exposure with ASD while reducing the influence of outliers, we assigned to each child the median pollutant concentration for his or her quintile and conducted logistic regression with these concentrations entered as a continuous independent variable. To test for sex differences in the association of pollutant quintile with ASD, we multiplied a continuous term for pollutant quintile (1–5) by an indicator of male sex and included this term in models with pollutant quintile, male sex, and demographic covariates. To adjust for multiple tests of significance, we calculated *p*-values adjusted for false discovery rate using the SAS Multtest procedure (SAS Institute Inc., Cary, NC).

Because individual metal concentrations were moderately or highly correlated in preliminary analyses [Pearson correlation coefficient range, 0.13–0.66; see Supplemental Material, Table S1 (http://dx.doi.org/10.1289/ehp.1206187)], we calculated an overall estimate of association with metal exposure by pooling ORs estimated for individual metals (antimony, arsenic, cadmium, chromium, lead, manganese, mercury, nickel), using a random-effects meta-analysis with the SAS Mixed procedure (Higgins et al. 2009). Additionally, we estimated associations between ASD and an overall measure of metal exposure that was derived by summing the quintile category score (1–5, with 1 representing the lowest quintile) for each metal (antimony, arsenic, cadmium, chromium, lead, manganese, mercury, nickel) to create an overall score with values ranging from 8 to 40.

We examined the association of the covariates with the overall metals metric and conducted additional analyses using this metric, examining the effects of state of residence, family income, smoking during pregnancy, HAP model year and urbanicity on the association between this metals metric and ASD [see Supplemental Material, Methods (http://dx.doi.org/10.1289/ehp.1206187)]. All models were adjusted for HAP year, tract median income, tract percent college educated, maternal age at birth, child’s year of birth, and maternal parents’ education. We did not adjust for family income or spouse/partner’s education in main analyses because they were measured after the child’s birth for most children, and the child’s ASD status may have affected income and educational attainment. Additionally, we did not adjust for smoking during pregnancy in the main analyses because 65 cases were missing smoking data (Table 1).

**Table 1:** Demographic characteristics of ASD cases and controls, children of the Nurses’ Health Study II, born 1987–2002 (*n* = 22,426).

Characteristic	ASD (*n *= 325)	No ASD (*n *= 22,101)	Missing data (*n* cases/*n* controls)
Maternal age at birth [years (mean ± SD)]	33.5 ± 4.3	32.6 ± 4.0	0/0
Year of child’s birth [median (range)]	1992 (1987–2002)	1990 (1987–2002)	0/0
Child’s sex			0/0
Female	46 (14.2)	11,092 (50.2)	0/0
Male	279 (85.9)	11,009 (49.8)	0/0
State of residence at birth			0/0
Michigan	40 (12.3)	2,585 (11.7)	0/0
New York	76 (23.4)	4,319 (19.5)	0/0
Ohio	37 (11.4)	2,843 (12.9)	0/0
Pennsylvania	43 (13.3)	3,260 (14.8)	0/0
All other states	129 (39.7)	9,094 (41.2)	0/0
HAP model year			0/0
1990	208 (64.0)	17,375 (78.6)	0/0
1996	81 (24.9)	3,350 (15.2)	0/0
1999	30 (9.2)	1,277 (5.8)	0/0
2002	6 (1.9)	99 (0.5)	0/0
Mother’s parents’ education (≤ high school)	158 (48.9)	9,416 (42.6)	0/0^*a*^
Tract % college educated [mean (range)]	33.4 (0.07–0.84)	32.3 (0.01–0.88)	0/0^*a*^
Tract median income ($) [mean (range)]	66,243 (24,000–200,000)^*b*^	67,051 (10,000–200,000)^*b*^	0/0^*a*^
Tract population density (persons/mi^2^) [mean (range)]	3,288 (0–92,000)^*b*^	1,003 (0–200,000)^*b*^	0/0^*a*^
Spouse/partner’s education (some graduate school)	96 (32.2)	6,458 (30.9)	27/1194
Smoking during index pregnancy	28 (10.8)	1,542 (7.0)	65/10
Family income, $50,000–74,000	78 (31.0)	4,615 (26.3)	73/4,523
Values are *n* (%) unless otherwise noted.^******^^***a***^Mean values were imputed for 140 persons missing parents’ education and 90 persons missing tract percent college educated, median income, and population density. ^***b***^Numbers in the range have been rounded to protect participants’ anonymity.			

To investigate further whether one or two pollutants were driving the association between correlated pollutants and ASD, we conducted analyses with diesel, lead, manganese, mercury, methylene chloride, and nickel—the pollutants most strongly associated with ASD based on tests of highest versus lowest quintile as well as linear trend—in a single model.

## Results

In cases (vs. controls), maternal age at birth was slightly older, year of birth was somewhat more recent, and maternal parents’ education somewhat lower, but residential census tract percent college educated and median income 6 years after the birth year were similar in the two groups (Table 1). The overall metals measure was positively associated with maternal parents’ education, tract median income, tract percent college educated, family income, and the 1990 HAP model year and negatively associated with year of birth and the 1996 and 1999 HAP model years [see Supplemental Material, Table S2 (http://dx.doi.org/10.1289/ehp.1206187)].

Adjusted ORs for ASD in association with the highest versus lowest quintile of lead, manganese, mercury, nickel, diesel particulate, methylene chloride, and the overall metal score were positive and statistically significant (*p* < 0.05), as was the pooled random-effects OR for metals (Table 2). ORs were positive but not statistically significant for the other exposures of *a priori* interest except quinoline. In sex-stratified models, adjusted ORs for the highest versus lowest quintile among boys were positive and statistically significant for diesel, methylene chloride, all metals except chromium and arsenic, and the overall metals score, as well as for the pooled OR for metals. Corresponding ORs for girls were near or below 1.0 for every exposure except manganese, mercury, diesel, methylene chloride, and quinoline, which had positive but not statistically significant associations with ASD (Table 2). We found a statistically significant linear sex-by-overall-metals-exposure interaction term (OR = 2.1; 95% CI: 1.8, 2.5), as well as statistically significant sex-by-pollutant interactions for nickel and antimony, and borderline significant interactions (*p* < 0.10) for cadmium, lead, and trichloroethylene. For antimony and trichloroethylene, higher pollutant concentration was associated with substantially lower ASD among girls, associations that are likely attributable to chance and may have accounted for the highly significant *p*-values for the pollutant-by-sex interaction terms. After adjustment for false discovery rate, lead, manganese, mercury, nickel, and methylene chloride remained statistically significantly associated with ASD for both sexes together. For boys, all pollutants that were statistically significant in initial analyses remained so after false-discovery-rate adjustment (i.e., diesel, methylene chloride, and all metals except chromium and arsenic).

**Table 2:** ORs of ASD by quintile of pollutant exposure, children of the Nurses’ Health Study II, born 1987–2002.^*a*^

	Quintile 1		Quintile 2		Quintile 3		Quintile 4		Quintile 5		Wald χ^2^ tests, *p*-values		
	Cases/controls (*n*)	OR (95% CI)	Cases/controls (*n*)	OR (95% CI)	Cases/controls (*n*)	OR (95% CI)	Cases/controls (*n*)	OR (95% CI)	Cases/controls (*n*)	OR (95% CI)	Trend	Q5 versus Q1	Sex-by-pollutant interaction
Pooled metals^*b*^
Both sexes		1.0 (Ref)		1.3 (1.1, 1.4)		1.3 (1.1, 1.5)		1.4 (1.2, 1.6)		1.5 (1.3, 1.7)	< 0.0001	< 0.0001	< 0.0001
Boys				1.2 (1.1, 1.4)		1.4 (1.2, 1.6)		1.4 (1.2, 1.6)		1.6 (1.4, 1.8)	< 0.0001	< 0.0001
Girls				1.4 (1.1, 2.0)		1.0 (0.7, 1.4)		1.1 (0.8, 1.6)		0.9 (0.7, 1.4)	0.34	0.74
Overall metals
Both sexes	57/4,577	1.0 (Ref)	76/4,420	1.4 (1.0, 2.1)	54/4,287	1.2 (0.8, 1.7)	81/4,731	1.8 (1.2, 2.6)	57/4,086	1.5 (1.0, 2.3)	0.02	0.04	< 0.0001
Boys	46/2,277		63/2,240	1.5 (1.0, 2.2)	48/2,188	1.3 (0.8, 1.9)	70/2,301	1.9 (1.3, 2.9)	52/2,086	1.7 (1.1, 2.6)	0.007	0.02
Girls	11/2,300		13/2,180	1.3 (0.6, 3.0)	6/2,099	0.7 (0.3, 2.0)	11/2,430	1.3 (0.5, 3.3)	5/2,000	0.8 (0.2, 2.4)	0.75	0.65
Antimony mean (μg/m^3^)		2 × 10^–5^		8 × 10^–5^		0.0002		0.0003		0.001
Both sexes	46/3,751	1.0 (Ref)	58/3,737	1.6 (1.0, 2.3)	34/3,770	0.9 (0.6, 1.5)	55/3,746	1.5 (1.0, 2.4)	51/3,747	1.4 (0.9, 2.2)	0.18	0.11	< 0.01
Boys	37/1,916		46/1,848	1.5 (1.0, 2.4)	27/1,937	0.9 (0.5, 1.5)	50/1,866	1.7 (1.1, 2.7)	49/1,870	1.7 (1.1, 2.7)	0.03	0.03
Girls	9/1,835		12/1,889	1.8 (0.7, 4.5)	7/1,833	1.1 (0.4, 31)	5/1,880	0.7 (0.2, 2.4)	2/1,877	0.3 (0.1, 1.5)	0.06	0.14
Arsenic mean (μg/m^3^)		3 × 10^–5^		0.0001		0.0002		0.0003		0.0008
Both sexes	64/4,417	1.0 (Ref)	72/4,414	1.4 (1.0, 2.0)	71/4,416	1.6 (1.1, 2.3)	58/4,428	1.4 (0.9, 2.1)	60/4,426	1.3 (0.9, 2.0)	0.32	0.18	0.45
Boys	53/2,220		60/2,184	1.4 (1.0, 2.1)	64/2,276	1.7 (1.1, 2.6)	50/2,189	1.5 (0.9, 2.3)	52/2,223	1.4 (0.9, 2.1)	0.24	0.15
Girls	11/2,197		12/2,230	1.4 (0.6, 3.4)	7/2,140	1.0 (0.4, 2.8)	8/2,239	1.2 (0.4, 3.3)	8/2,203	1.0 (0.3, 2.8)	0.83	0.97
Cadmium mean (μg/m^3^)		2 × 10^–5^		6 × 10^–5^		0.0001		0.0002		0.0006
Both sexes	55/4,434	1.0 (Ref)	66/4,416	1.3 (0.9, 1.9)	68/4,418	1.4 (1.0, 2.1)	67/4,419	1.5 (1.0, 2.2)	69/4,414	1.5 (1.0, 2.1)	0.06	0.05	< 0.10
Boys	45/2,228		54/2,196	1.3 (0.9, 2.0)	59/2,244	1.5 (1.0, 2.3)	58/2,193	1.5 (1.0, 2.4)	63/2,231	1.6 (1.1, 2.4)	0.02	0.02
Girls	10/2,206		12/2,220	1.3 (0.5, 3.1)	9/2,174	1.1 (0.4, 3.0)	9/2,226	1.1 (0.4, 3.0)	6/2,183	0.7 (0.3, 2.1)	0.54	0.57
Chromium mean (μg/m^3^)		0.0001		0.0004		0.0007		0.0012		0.0037
Both sexes	50/4,175	1.0 (Ref)	54/4,171	1.1 (0.7, 1.6)	52/4,174	1.0 (0.7, 1.5)	59/4,165	1.1 (0.7, 1.6)	80/4,139	1.4 (0.9, 2.0)	0.13	0.12
Boys	42/2,110		47/2,110	1.1 (0.7, 1.7)	46/2,067	1.1 (0.7, 1.7)	51/2,090	1.1 (0.7, 1.7)	70/2,097	1.4 (0.9, 2.1)	0.11	0.09
Girls	8/2,065		7/2,061	0.9 (0.3, 2.5)	6/2,107	0.7 (0.2, 2.0)	8/2,075	0.9 (0.3, 2.5)	10/2,042	1.0 (0.4, 2.8)	0.91	0.97	0.63
Lead mean (μg/m^3^)		0.0008		0.0020		0.0034		0.0052		0.015
Both sexes	55/4,431	1.0 (Ref)	55/4,431	1.1 (0.8, 1.7)	70/4,416	1.6 (1.1, 2.3)	71/4,415	1.7 (1.1, 2.4)	74/4,408	1.6 (1.1, 2.3)	0.003	0.02	< 0.10
Boys	44/2,215		43/2,232	1.1 (0.7, 1.7)	65/2,238	1.8 (1.2, 2.7)	61/2,173	1.8 (1.2, 2.7)	66/2,234	1.7 (1.2, 2.6)	0.001	0.008
Girls	11/2,216		12/2,199	1.3 (0.5, 3.0)	5/2,178	0.6 (0.2, 1.8)	10/2,242	1.3 (0.5, 3.2)	8/2,174	0.9 (0.4, 2.4)	0.84	0.86
Manganese mean (μg/m^3^)		0.0006		0.0016		0.0027		0.0041		0.011
Both sexes	62/4,423	1.0 (Ref)	67/4,417	1.3 (0.9, 1.8)	65/4,421	1.3 (0.9, 1.9)	59/4,426	1.3 (0.9, 1.9)	72/4,414	1.5 (1.1, 2.2)	0.03	0.02	0.47
Boys	53/2,210		53/2,218	1.1 (0.8, 1.7)	60/2,215	1.4 (0.9, 2.0)	50/2,244	1.2 (0.8, 1.8)	63/2,205	1.5 (1.0, 2.3)	0.04	0.03
Girls	9/2,213		14/2,199	2.1 (0.9, 5.0)	5/2,206	0.9 (0.3, 2.8)	9/2,182	1.8 (0.6, 4.9)	9/2,209	1.6 (0.6, 4.2)	0.55	0.36
Mercury mean (μg/m^3^)		0.0006		0.0016		0.0017		0.0019		0.0027
Both sexes	89/4,392	1.0 (Ref)	61/4,424	1.6 (0.9, 2.6)	49/4,439	1.3 (0.8, 2.3)	53/4,431	1.5 (0.9, 2.6)	73/4,415	2.0 (1.2, 3.3)	0.02	0.008	0.18
Boys	77/2,173		47/2,250	1.3 (0.7, 2.2)	42/2,224	1.2 (0.7, 2.1)	46/2,200	1.4 (0.8, 2.5)	67/2,245	1.9 (1.1, 3.3)	0.008	0.02
Girls	12/2,219		14/2,174	4.7 (1.2, 18.0)	7/2,215	2.7 (0.6, 11.5)	7/2,231	2.8 (0.7, 11.9)	6/2,170	2.2 (0.5, 9.3)	0.87	0.26
Nickel mean (μg/m^3^)		0.0004		0.0012		0.0024		0.0045		0.0159
Both sexes	58/4,427	1.0 (Ref)	68/4,418	1.3 (0.9, 1.9)	71/4,413	1.6 (1.1, 2.2)	64/4,419	1.5 (1.0, 2.2)	64/4,424	1.7 (1.1, 2.5)	0.01	0.02	< 0.05
Boys	46/2,203		57/2,234	1.4 (0.9, 2.0)	60/2,249	1.6 (1.1, 2.4)	58/2,232	1.7 (1.1, 2.6)	58/2,174	1.9 (1.2, 2.9)	0.004	0.004
Girls	12/2,224		11/2,184	1.1 (0.5, 2.5)	11/2,164	1.2 (0.5, 3.0)	6/2,187	0.7 (0.3, 2.1)	6/2,250	0.7 (0.2, 2.2)	0.48	0.58
Diesel particulate mean (µg/m^3^)		0.60		1.06		1.48		2.00		4.40
Both sexes	18/953	1.0 (Ref)	21/948	1.1 (0.6, 2.2)	24/947	1.3 (0.7, 2.5)	21/947	1.2 (0.6, 2.5)	33/931	2.0 (1.0, 4.0)	0.05	0.04	0.12
Boys	13/451		16/451	1.2 (0.6, 2.5)	19/493	1.4 (0.6, 2.9)	21/468	1.7 (0.8, 3.6)	28/462	2.3 (1.1, 4.9)	0.02	0.04
Girls	5/502		5/497	1.2 (0.3, 4.3)	5/454	1.4 (0.4, 5.3)	0/479	Not estimable^*c*^	5/469	1.5 (0.3, 7.0)	0.98	0.58
Methylene chloride mean (μg/m^3^)		0.15		0.23		0.32		0.43		0.98
Both sexes	53/4,436	1.0 (Ref)	56/4,429	1.4 (1.0, 2.1)	76/4,409	2.0 (1.3, 2.9)	67/4,417	1.6 (1.1, 2.4)	73/4,410	1.8 (1.2, 2.7)	0.008	0.004	0.62
Boys	45/2,244		46/2,209	1.4 (0.9, 2.2)	66/2,182	2.0 (1.3, 3.1)	60/2,283	1.7 (1.1, 2.6)	62/2,174	1.8 (1.2, 2.8)	0.006	0.006
Girls	8/2,192		10/2,220	1.7 (0.6, 4.5)	10/2,227	1.7 (0.6, 4.5)	7/2,134	1.1 (0.4, 3.4)	11/2,236	1.5 (0.5, 4.2)	0.75	0.43
Quinoline mean (μg/m^3^)		0						4 × 10^–7^		0.0001
Both sexes	166/13,885	1.0 (Ref)		—^*d*^		—^*d*^	85/3,810	0.8 (0.5, 1.4)	74/4,406	1.0 (0.7, 1.4)	0.76	0.89	0.10
Boys	148/6,967						70/1,880	0.7 (0.4, 1.3)	61/2,245	0.9 (0.6,1.3)	0.84	0.68
Girls	18/6,918						15/1,930	1.8 (0.5, 6.2)	13/2,161	1.9 (0.8, 4.3)	0.16	0.14
Styrene mean (μg/m^3^)		0.007		0.020		0.039		0.066		0.185
Both sexes	48/4,441	1.0 (Ref)	78/4,411	1.6 (1.1, 2.4)	54/4,429	1.0 (0.7, 1.6)	78/4,403	1.6 (1.1, 2.4)	67/4,417	1.4 (1.0, 2.1)	0.18	0.09	0.30
Boys	39/2,256		66/2,240	1.7 (1.1, 2.6)	47/2,167	1.1 (0.7, 1.7)	68/2,181	1.7 (1.1, 2.6)	59/2,248	1.5 (1.0, 2.3)	0.14	0.07
Girls	9/2,185		12/2,171	1.4 (0.6, 3.6)	7/2,262	0.7 (0.3, 2.0)	10/2,222	1.3 (0.5, 3.2)	8/2,169	1.1 (0.9, 1.3)	0.93	0.92
Trichloroethylene mean (μg/m^3^)		0.06		0.13		0.22		0.33		0.65
Both sexes	84/4,420	1.0 (Ref)	68/4,390	1.5 (1.0, 2.2)	55/4,433	1.4 (0.9, 2.2)	68/4,421	1.8 (1.2, 2.8)	50/4,437	1.3 (0.8, 2.1)	0.31	0.26	< 0.10
Boys	72/2,194		52/2,223	1.3 (0.9, 2.0)	46/2,199	1.4 (0.8, 2.2)	62/2,235	1.9 (1.2, 3.0)	47/2,241	1.4 (1.1, 2.3)	0.12	0.19
Girls	12/2,226		16/2,167	2.5 (1.1, 5.9)	9/2,234	1.8 (0.6, 5.2)	6/2,186	1.3 (0.4, 4.4)	3/2,196	0.6 (0.1, 2.7)	0.26	0.52
Vinyl chloride mean (μg/m^3^)		0.0004		0.0021		0.0067		0.0180		0.0898
Both sexes	57/4,432	1.0 (Ref)	68/4,417	1.3 (0.9, 1.8)	75/4,410	1.5 (1.0, 2.2)	62/4,425	1.4 (0.9, 2.0)	63/4,417	1.2 (0.8, 1.8)	0.33	0.49	0.92
Boys	47/2,236		60/2,217	1.4 (0.9, 2.0)	66/2,206	1.6 (1.1, 2.4)	54/2,199	1.5 (1.0, 2.3)	52/2,234	1.2 (0.7, 1.9)	0.32	0.44
Girls	10/2,196		8/2,200	0.9 (0.3, 2.2)	9/2,204	1.0 (0.4, 2.5)	8/2,226	1.0 (0.4, 2.8)	11/2,183	1.0 (0.3, 3.0)	0.93	0.98
Ref, reference. ^***a***^Quintiles of pollutant exposure are based on the entire sample. Models were adjusted for maternal age at birth, year of birth, maternal parents’ education, census tract median income, census tract percent college educated, and HAP model year. Models not stratified by sex were adjusted for sex. Antimony was not available in the 1996 model year, chromium was not available in the 1999 model year, and diesel was not available in the 1990 model year. ^***b***^Estimates for the association of pooled metals with ASD were calculated using a random-effects meta-analysis with the SAS Mixed ­procedure. ^***c***^Not estimable due to sparseness of cases in this cell. ^***d***^The distribution of quinoline did not permit creation of quintiles, so we present tertiles: quintile 1 indicates tertile 1 (reference), quintile 4 indicates tertile 2, and quintile 5 indicates tertile 3.													

**Figure 1:**
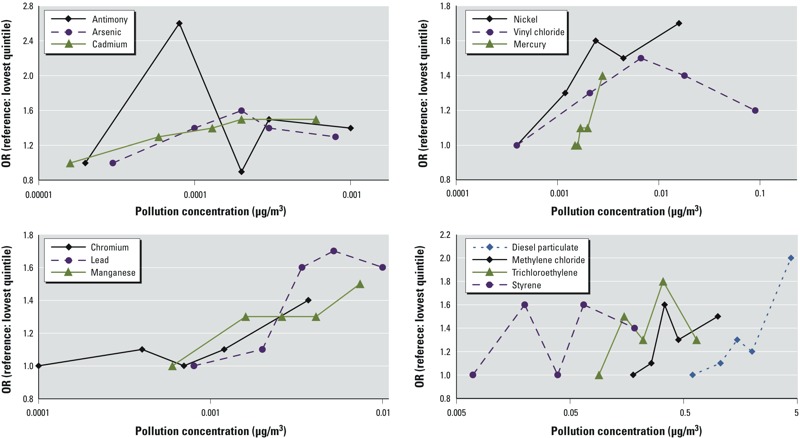
OR of ASD for each quintile of pollutant (lowest quintile is the reference) by pollutant concentration, for children of the Nurses’ Health Study II (*n* = 22,101 controls, *n* = 325 cases). Details on ORs and concentrations for each quintile of each chemical can be found in Table 2.

Cadmium, diesel, lead, manganese, mercury, methylene chloride, and nickel showed roughly linear dose–response relationships between concentration and OR of ASD (Figure 1). Of these, the test of linear trend in models with each observation assigned the median of its quintile was statistically significant for all except cadmium (*p* = 0.06). The pooled metals metric was significant as well (Table 2). In models restricted to boys, linear trends were steeper with smaller *p*-values. Adjusted for false discovery rate, no linear trend tests were significant at *p* < 0.05 for both sexes together. For boys, linear trends of all metals except for cadmium were statistically significantly associated with ASD after adjustment for false discovery rate, as were diesel and methylene chloride (Table 2). We did not find meaningful differences in the relationship between the overall metals metric and ASD in models accounting for birth state, family income, spouse/partner’s education, smoking during pregnancy, HAP model year, or urbanicity [see Supplemental Material, Table S3 (http://dx.doi.org/10.1289/ehp.1206187)].

In a multipollutant model including the pollutants most strongly associated with ASD (lead, manganese, mercury, methylene chloride, and nickel), ORs for all pollutants were attenuated compared with single-pollutant models, with the OR for manganese falling below 1.0 [see Supplemental Material, Table S4 (http://dx.doi.org/10.1289/ehp.1206187)]. Estimates for mercury (OR = 1.5; 95% CI: 0.8, 2.9) and methylene chloride (OR = 1.4; 95% CI: 0.9, 2.3) were largest. In models restricted to boys, ORs for lead, mercury, nickel, and methylene chloride were largest. In the subsample with data on diesel (HAP model years 1996, 1999, and 2002; *n* controls = 4,726, *n* cases = 117), adding the other chemicals to the model substantially attenuated the OR for diesel (both sexes, OR = 1.2; 95% CI: 0.5, 2.9; boys only, OR = 1.5; 95% CI: 0.6, 4.1). The high correlation between pollutants, however, could contribute to lowered OR. The correlation between mercury and diesel, for example, was particularly high (Pearson’s *r* = 0.84).

To examine how the pollutants selected *a priori* compared with other pollutants in their relationship with ASD, we calculated ORs of ASD for the highest versus lowest quintile of exposure for each pollutant in the U.S. EPA database, adjusted for HAP year, tract median income, tract percent college educated, maternal age at birth, child’s year of birth, and maternal parents’ education [see Supplemental Material, Table S5 (http://dx.doi.org/10.1289/ehp.1206187)]. We compared the likelihood of pollutants selected *a priori* versus not selected *a priori* being associated with elevated of ASD by dividing the number of statistically significant pollutants by total pollutants in each of these groups. Too few localities reported pollutant concentration or the distribution of the pollutant was not adequate for model convergence for 18 of 198 pollutants. Of the remaining 180, 26 had statistically significant positive associations with ASD for the highest versus lowest quintile, whereas 1 had a statistically significant negative association. Of the 26 with significant positive associations, 7 were among the 14 pollutants selected *a priori*. Thus the pollutants identified *a priori* were more than four times as likely to be statistically significantly positively associated with elevated ASD than were the pollutants not identified *a priori*.

**Figure 2:**
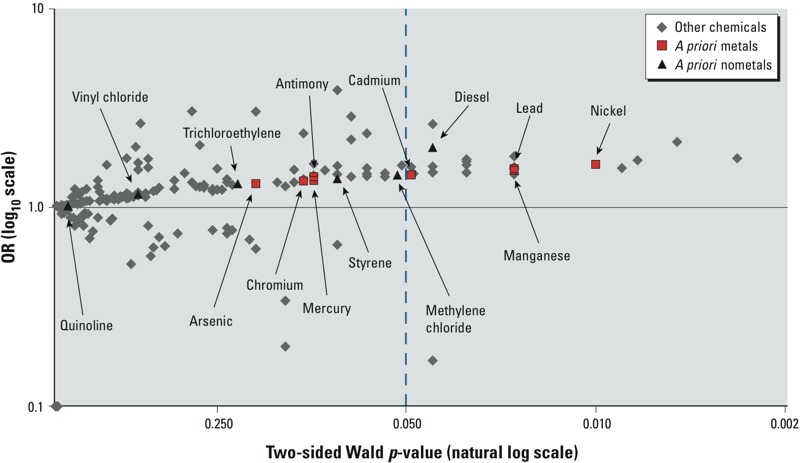
Association of ASD with air pollutant concentration, highest quintile versus lowest quintile ORs by Wald two-sided *p*-value, children of the Nurses’ Health Study II (*n* = 22,101 controls, *n* = 325 cases).

To graphically represent the distribution of statistical test results among the many pollutants in the U.S. EPA data, we plotted ORs (*y*-axis, log_10_ scale) and *p*-values (*x*-axis, natural log scale) for the highest concentration quintile versus the lowest concentration quintile for all pollutants (Figure 2). If the associations of ASD with the pollutants were attributable to random noise, we would expect a symmetrical distribution around 1, with a wider distribution of ORs as the *p*-values got smaller. Figure 2 shows roughly this type of symmetrical distribution at *p*-values ≥ 0.05, but at *p*-values < 0.05 there is a clear predominance of positive ORs that are not mirrored by negative ORs. Figure 2 therefore graphically suggests that the data contain some positive signal for a risk factor for ASD. Among pollutants not selected *a priori*, five were associated with ASD with a high level of statistical significance [i.e., *p* < 0.01; see Supplemental Material, Table S5 (http://dx.doi.org/10.1289/ehp.1206187)]: beryllium, acetonitrile, tetrachloroethylene (“perc”), 1,2,4-trichlorobenzene, and ethylene dichloride.

## Discussion

To our knowledge, our study is the first to examine the association between air pollution and ASD across the United States. Our study adds to existing evidence that maternal exposure to air pollution in the perinatal period may increase risk for ASD in children. We observed significant positive linear trends between pollutant concentration and ASD for diesel particulate matter, lead, manganese, methylene chloride, mercury, and nickel. The positive trend for the pooled metals metric and ASD was highly significant and displayed the most regularly monotonic linear dose response of all exposures examined. Additionally, of all pollutants in the data set, the highest quintiles of diesel and mercury were most strongly associated with ASD, and, unlike most pollutants examined, these were positively associated with ASD in both boys and girls. The association between ASD and exposure to diesel particulate matter is particularly notable: Data on diesel exposure were available for only 4,843 respondents because diesel was not reported in the 1990 HAP model year.

Possibly because of the high correlation of pollutants, the use of modeled rather than actual exposures, or limited power, we were unable to identify one or two pollutants as being most responsible for driving associations with ASD. Studies with a larger number of ASD cases or with pollution exposure measured more precisely may be able to provide stronger evidence for the etiologic role of a specific pollutant. Alternatively, several pollutants may be associated with ASD through their neurotoxic, mutagenic, or other effects.

Given the small number of girls with ASD (*n* = 46), the sex differences in our results must be interpreted with caution. It is possible that the few pollutants that showed a positive association with ASD for both boys and girls are more likely candidates to be causally associated with ASD. On the other hand, most of the pollutants we examined showed a much more robust association among boys—the sex-by-pooled-metals interaction term was large and statistically significant—and sex-specific etiological subtypes of ASD have been suggested by prior findings. Specifically, the male:female sex ratio is significantly higher in ASD without comorbid mental retardation compared with ASD with mental retardation (Fombonne 2007), and Rett syndrome, a rare form of autism, occurs only in girls. Metals, traffic-related pollution (e.g., diesel), and styrene are neurotoxicants, induce inflammatory responses in humans and animals, (Block and Calderón-Garcidueñas 2009; Diodovich et al. 2004; Lee et al. 2011), and lead to oxidative stress in *in vitro* studies and *in vivo* animal studies (Valko et al. 2005)—processes that have been implicated in autism (Vargas et al. 2005). Greater innate vulnerability to ASD in boys versus girls, as reflected in the greater prevalence of ASD in boys, may mean that the neurotoxic and inflammatory effects of these air pollutants are more likely to cause boys to cross a biological or behavioral threshold into ASD. Additionally, metals may have sex-specific effects on social behavior through altered dopamine function (Curtis et al. 2010).

Five prior studies have examined air pollution and ASD, including two studies that used the same U.S. EPA models as we did to estimate exposure to pollutants. The first study, of the San Francisco Bay area, found elevated ASD associated with exposure to metals (cadmium, mercury, and nickel), chlorinated solvents (methylene chloride, trichloroethylene, and vinyl chloride), and diesel (ASD cases, *n* = 284; controls, *n* = 657, identified through the California autism surveillance system) (Windham et al. 2006). A second study in North Carolina and West Virginia, which compared children with ASD, identified from the Autism and Developmental Disabilities Monitoring Network (*n* = 383), with children with speech and language delays (*n* = 2,829), found significantly elevated ASD for 17 of 35 pollutants in unadjusted models, including diesel, mercury, nickel, and beryllium, but not lead or methylene chloride. However, risk was attenuated in models adjusted for urbanicity, socioeconomic status, and other covariates. No pollutants were statistically significantly associated with increased ASD in these adjusted models (Kalkbrenner et al. 2010). However, because urbanicity may be a proxy for pollutant concentration, including it in models may effectively capture some of an association that in reality should be attributed to pollutants, thus biasing results. In our analyses, adjusting for population density very slightly attenuated associations of pollutants with ASD. To the extent that any pollutant was also associated with speech and language delays, associations with ASD would be attenuated because of the use of such children as controls. In semi-Bayes models with multiple pollutants as predictors, designed to determine which among many correlated pollutants were associated with ASD, associations with styrene and quinoline just reached significance (Kalkbrenner et al. 2010). Similarly, in models including multiple pollutants we found that ORs for each pollutant were attenuated compared with single-pollutant models, and no longer statistically significant.

Mother’s residential proximity to a major freeway during pregnancy has also been associated with ASD in a study including 304 ASD cases recruited through the California Department of Developmental Services and 259 sex-, age-, and geographically matched controls (Volk et al. 2011). Distance to a freeway may reflect approximate exposure to pollutants from traffic. Given our finding that diesel concentration was associated with ASD, this study and ours, which used very different measures, together suggest that traffic-related air pollution may increase the risk of ASD. Finally, a study of Texas school districts found that environmentally released mercury in a county was associated with higher autism prevalence among students in that county (Palmer et al. 2009), but these results were not replicated (Lewandowski et al. 2009). Relying on school district data for ASD case status could introduce bias related to differential rates of identification of children for special education by school district. This is of particular concern because the associations were substantially reduced in analyses that attempted to account for possible bias by adjusting for special education rates excluding autism.

Prior studies have on the whole not examined sex differences in the relationship between ASD and air pollutants (Larsson et al. 2009; Lewandowski et al. 2009; Palmer et al. 2009; Roberts et al. 2007; Volk et al. 2011). Kalkbrenner et al. (2010) found a suggestion of sex differences in the relationship between ASD and several pollutants, although they did not find associations consistently higher in boys versus girls. Associations of ASD with styrene, butadiene, and tetrachloroethane were elevated for boys and not girls, whereas the association of ASD with mercury compounds were elevated for girls and not boys (Kalkbrenner et al. 2010). However, this study and ours had limited power to test for sex differences because few girls with ASD were included.

Our study has several limitations. The U.S. EPA air pollution prediction models (U.S. EPA 2011) provide only approximate measures of pollutant exposures, and the prediction modeling technique differed in the four assessment models. Ideally, pollution exposure would be measured individually to account for variation in exposure due to time spent outdoors, commuting, indoor exposure, seasonal fluctuation, and neighborhood-level variations in pollutants. However, validation studies of U.S. EPA models have found them to reflect relative exposure fairly accurately (Payne-Sturges et al. 2004; State of New Jersey Department of Environmental Protection 2011; U.S. EPA Scientific Advisory Board 2001). An important caveat is that for pollutants for which the primary source is indoor as opposed to outdoor (e.g., from lead paint, drinking water, or household chemicals vs. from traffic or industry emissions), U.S. EPA exposures may not accurately measure relative exposure. For example, personal exposure to styrene, chloroform, and tetrachloroethylene (perc) were underestimated substantially more than exposure to other air pollutants in a validation study, most likely because exposures were predominantly from indoor sources (Payne-Sturges et al. 2004).

Additionally, we determined nurses’ residential location during the perinatal period from the year of birth of the child and the address reported when the biennial questionnaire was returned. Thus, for nurses who moved residences around the time of the pregnancy, residential location during and immediately following pregnancy may be incorrect. This could lead to additional exposure misclassification, particularly for specific gestational stages, which may be relevant for ASD risk (Atladóttir et al. 2010; Kinney et al. 2008; Roberts et al. 2007). We relied on the nurse respondents for ascertainment of case status. However, in a subsample who were administered the ADI-R by telephone, we found excellent agreement with maternal report. Finally, we had information on family income only after the birth of the child. Income before the birth could be associated with unmeasured ASD risk factors or with ASD ascertainment, and failure to adjust for this may have biased estimates.

Despite the limitations, there are several strengths of our study that lend weight to the findings. Our study used a large national sample rather than a sample restricted to one or two states, so our participants were exposed to a wide range of pollutant concentrations. As nurses, the mothers in our study are likely to have more homogeneous health-seeking behavior than other samples studied, because of their shared occupation as health workers. Thus, it seems likely that ASD reporting would be more homogeneous than in more general population samples in which there may be underascertainment of cases in families with low income or low education (Kalkbrenner et al. 2012). We also conducted analyses adjusted for a range of socioeconomic indicators—which could also be associated with air pollution—to further guard against ascertainment bias. Thus, we believe that the associations we found between pollutants and ASD were unlikely to have been caused by differences in ascertainment of ASD.

Further studies examining personal exposure to toxicants during gestation and concentrations of toxicants in the blood of newborns may permit the identification of specific agents that increase risk for ASD. Some states and national governments collect newborn blood, either to bank cord blood for therapeutic use or as part of a newborn screening program (Michigan Neonatal Biobank 2011). Stored blood can be used to assay pollutants (e.g., lead, mercury) and could be linked to ASD data sets to enable large-scale case–control studies (McCann 2011; Spliethoff et al. 2008). Given results of our present study, which strongly support previous evidence of associations between air pollution exposure and ASD, such studies are warranted.

## Correction

In the manuscript originally published online, the units given for diesel particulate concentration were incorrect in Table 2 and Figure 1 and in Supplemental Material, Table S1: Grams per cubic meter (g/m^3^) should have been micrograms per cubic meter (µg/m^3^). In addition, the number of controls given in Table 2 for boys and girls were inadvertently reversed for each exposure and quintile.

These errors have been corrected here.

## Supplemental Material

(426 KB) PDF
