# Erratum: “Perinatal Air Pollutant Exposures and Autism Spectrum Disorder in the Children of Nurses’ Health Study II Participants”

**DOI:** 10.1289/ehp.122-A152

**Published:** 2014-06-01

Roberts et al. have reported errors in their article “Perinatal Air Pollutant Exposures and Autism Spectrum Disorder in the Children of Nurses’ Health Study II Participants” [Environ Health Perspect 121:978–984 (2013); http://dx.doi.org/10.1289/ehp.1206187]. In hazardous air pollutant (HAP) data used in their paper, U.S.-wide background concentrations were not added to the concentration estimates for 13 HAPs [benzene, carbon tetrachloride, chloroform, ethylene dibromide, ethylene dichloride, formaldehyde, hexachlorobenzene, mercury, methylene chloride, polychlorinated biphenyls, tetrachloroethylene (perchloroethylene), trichloroethylene, and xylene] in the 1996 HAP model year. As a result, effect estimates reported for quintiles of these 13 HAPs formed using all 4 HAP years were in fact effect estimates for somewhat different percentile comparisons; for example, results shown for the comparison of the highest versus lowest quintile of mercury were actually closer to the highest quartile versus the lowest 5%. Results for mercury, methylene chloride, and tetrachloroethylene were incorrect in Figure 1 and Table 2, and several values were incorrect in Figure 2. Because mercury was a constituent (one of eight) of the pooled and overall metals measures, corrected results related to pooled metals and overall metals quintiles in Table 2 are slightly different, as are results in Supplemental Material. In addition, the median—rather than the mean—tract population density was inadvertently entered for those without autism spectrum disorder in Table 1; the correct mean value is 2,578 persons/mi^2^.

The changes largely do not affect the conclusions of the study, except that the results for mercury and methylene chloride for both sexes combined did not quite reach statistical significance at *p* < 0.05. In addition, in multipollutant models, the odds ratio for lead became the strongest, rather than mercury and methylene chloride, as the authors reported in the original version of the paper.

The article, including tables and figures, as well as the Supplemental Material have been corrected online.

The authors regret the errors.

