# Supplementary material for: Perinatal Air Pollutant Exposures and Autism Spectrum Disorder in the Children of Nurses’ Health Study II Participants
Source: Environ Health Perspect. 2013 Jun 18;121(8):978–84. doi: 10.1289/ehp.1206187 (PMC3734496; doi:10.1289/ehp.1206187)
Supplement: (426 KB) PDF [file ehp.1206187.s001.pdf]

Environmental Health Perspectives doi:10.1289/ehp.1206187

Supplemental Material

Title: Perinatal air pollutant exposures and autism spectrum disorder.

Authors: Andrea L. Roberts, Kristen Lyall, Jaime E. Hart, Francine Laden, Allan C. Just,

Jennifer F. Bobb, Karestan C. Koenen, Alberto Ascherio, and Marc G. Weisskopf

Contents

Supplemental Material, Methods 3

Supplemental Material, Results 3

Supplemental Material, Table S1: Pollutant concentration and Pearson correlation coefficients, children of the Nurses' Health Study II, (n=22,426) 5

Supplemental Material, Table S2: Potential confounders by quintiles of overall metals variable, children of the Nurses' Health Study II (n=22,426) 6

Supplemental Material, Table S3: Odds ratio of autism spectrum disorder with overall metals measure, sensitivity analyses, children of the Nurses' Health Study II 7

Supplemental Material, Table S4: Odds ratio [highest versus lowest quintile (95% confidence interval)] of autism spectrum disorder in multiple-pollutant and single-pollutant models, children of the Nurses' Health Study II, born 1987-2002 8

Supplemental Material, Table S5: Odds ratio of autism in child, highest pollutant concentration quintile versus lowest quintile exposure in child's birth year, children of the Nurses' Health Study II (n=22,442), listed by *P*-value. 9

Supplemental Material, Methods

Analysis

To explore possible ascertainment bias by state, we restricted to participants from the 6 states with the most respondents (California, Ohio, New York, Pennsylvania, Texas, and Michigan) and entered state as a categorical independent variable in the model. We added maternal smoking during pregnancy to models to see if smoking status confounded the association between metal exposure and ASD. To further explore possible ascertainment bias from socioeconomic status, we included family income and partner's educational attainment in models. We did not include these measures in the main analyses as child's ASD status may have affected family income and educational attainment. Additionally, 23% of respondents did not provide income information. We investigated possible bias caused by using different air pollution assessment models for different birth years by analyzing data restricted to respondents with pollution data from either the 1990 pollution assessment or the 1996 pollution assessment (separately), which included respectively 78.4% (n=17,583) and 15.3% (n=3,431) of the children. To examine possible ascertainment bias by urbanicity, we added census-tract-level population density to the models and examined associations of metals with ASD in the highest and lowest quartiles of population density.

Supplemental Material, Results

In a model restricted to the six states with the most respondents and adjusted for individual states, the odds ratio of ASD for the highest quintile of exposure versus the lowest for metals was slightly larger (OR=2.2, 95% CI=1.3, 3.8) than in the base model including all states (OR=1.6, 95% CI=1.1, 2.4, Table S3). We found no appreciable change in the estimated

association between the overall metals metric and ASD in additional analyses with models that included smoking during pregnancy, family income, or mother's partner's education.

In analyses restricted to the 1990 or 1996 air pollution prediction models, the odds ratio for the highest versus lowest quintile of overall metals measure was similar, with overlapping confidence intervals, though somewhat lower in the 1990 model (OR=1.4, 95% CI=0.9, 2.4) and higher in the 1996 model (OR=1.9, 95% CI=0.8, 4.2) compared with the analyses including all years. In the analyses adjusted for population density, the overall metals measure was similarly associated with ASD (OR=1.6, 95% CI=1.0, 2.4). We additionally examined all the pollutants selected *a priori* in models adjusted for census-tract-level population density. In general, estimates were very slightly attenuated: lead, manganese, and nickel remained statistically significantly associated with ASD in both sexes combined. The mercury and methylene chloride linear trend associations with ASD (p=0.05 and p=0.08, respectively) were also attenuated (to p=0.10 and 0.14, respectively). Population density and metals concentration were associated (continuous measures, Pearson correlation coefficient = 0.26; agreement between quintiles, weighted kappa = 0.37). In models stratified by population density, the odds ratio for highest quintile of metals concentration was attenuated among respondents who lived in the least dense quartile of census tracts (OR=1.1, 95% CI=0.4, 3.4) and elevated among respondents who lived in the most dense quartile of census tracts (OR=10.1, 95% CI = 1.3, 79.2).

Supplemental Material, Table S1: Pollutant concentration and Pearson correlation coefficients, children of the Nurses' Health Study II, (n=22,426)

	Sb	As	Cd	Cr	DPM	Pb	Mn	Hg	MC	Ni	QN	Styrene	TCE	VC
Concentration														
Range (ug/m^3,)	0 - .02	0 – .05	0 – .03	2x10^{-6} – .06	0 – 139.8	0- .65	0 - .62	1x10^{-6} - .08	6x10^{-4} – 41.9	0 - .42	0 - .007	0 – 7.3	5x10^{-3} – 5.0	0 – 3.2
Mean (SD)	3x10^{-4} (7x10^{-4})	2x10^{-4} (5x10^{-4})	2x10^{-4} (5x10^{-4})	.001 (.002)	1.9 (3.0)	.005 (.01)	.004 (.008)	.002 (.001)	.4 (.7)	.005 (.01)	2x10^{-5} (1x10^{-4})	.06 (.1)	.3 (.3)	.02 (.06)
Correlation														
Antimony	1.0	0.46	.27	.20	.03	.20	.21	.57	.08	.66	-.02	.17	.30	.11
Arsenic		1.0	.35	.23	.08	.22	.18	.19	.08	.41	.08	.15	.24	.10
Cadmium			1.0	.35	.19	.21	.15	.32	.10	.24	.01	.14	.20	.09
Chromium				1.0	.31	.19	.24	.25	.10	.22	.12	.12	.19	.05
Diesel					1.0	.36	.08	.91	.34	.28	.01	.33	.40	-.01
Lead						1.0	.13	.22	.07	.17	.00	.10	.20	.05
Manganese							1.0	.19	.06	.38	.01	.11	.21	.07
Mercury								1.0	.16	.32	-.01	.20	.29	.08
Methylene Chloride									1.0	.12	.00	.14	.27	.10
Nickel										1.0	-.01	.21	.37	.15
Quinoline											1.0	.01	-.03	.00
Styrene												1.0	.26	.80
Trichloro-ethylene													1.0	.18
Vinyl Chloride														1.0

Sb = antimony, As = arsenic, Cd = cadmium, Cr = chromium, DPM = diesel particulate matter, Pb = lead, Mn = manganese, Hg = mercury, MC = methylene chloride, Ni = nickel, QN = quinoline, TCE = trichloroethylene, VC = vinyl chloride

Supplemental Material, Table S2: Potential confounders by quintiles of overall metals variable, children of the Nurses' Health Study II (n=22,426)

	Q1: Lowest concentration	Q2	Q3	Q4	Q5: Highest concentration
Cases / controls, N	50 / 4300	75 / 4506	58 / 4453	82 / 4718	60 / 4124
Maternal age at birth, years, mean ± standard deviation	33.0 ± 4.4	32.6 ± 4.1	32.6 ± 4.0	32.4 ± 3.8	32.6 ± 3.8
Year of birth, median (range)	1991 (1987-2001)	1990 (1987-2001)	1990 (1987 - 2002)	1990 (1987 - 2001)	1990 (1987-2000)
Sex, N (%)					
Female	2173 (50.0)	2238 (48.9)	2206 (48.9)	2414 (50.3)	2024 (48.4)
Male	2177 (50.1)	2343 (51.2)	2305 (51.1)	2386 (49.7)	2160 (51.6)
State of residence, N (%)					
Michigan	599 (13.8)	596 (13.0)	418 (9.3)	413 (8.6)	599 (14.3)
New York	730 (16.8)	733 (16.0)	767 (17.0)	897 (18.7)	1268 (30.3)
Ohio	282 (6.5)	466 (10.2)	581 (12.9)	895 (18.7)	656 (15.7)
Pennsylvania	522 (12.0)	550 (12.0)	786 (17.4)	730 (15.2)	715 (17.1)
All other states	2217 (51.0)	2236 (48.8)	1959 (43.4)	1865 (38.9)	946 (22.6)
HAP model year, N (%)					
1990	2759 (63.4)	3455 (75.4)	3409 (75.6)	4181 (87.1)	3779 (90.3)
1996	910 (20.9)	743 (16.2)	914 (20.3)	526 (11.0)	338 (8.1)
1999	662 (15.2)	327 (7.1)	164 (3.6)	87 (1.8)	67 (1.6)
2002	19 (0.4)	56 (1.2)	24 (0.5)	6 (0.1)	0 (0)
Mother's parents' education, high school or less, N (%)	2006 (46.1)	1975 (43.1)	1862 (41.3)	1973 (41.1)	1759 (42.0)
Tract % college educated, mean (range)†	24.0 (2 - 88)	30.6 (2 - 88)	34.2 (3 - 86)	36.0 (1 - 88)	36.7 (1 - 86)
Tract median income, $, mean (range)†	54,693 (14,900-200,000)	63,039 (14,100-200,000)	68,521 (11,000-200,000)	70,908 (13,600-200,000)	74,013 (9,700-200,000)
Tract population density, persons/mile^2, mean (range)†	699 (0-26,300)	1,233 (0 - 27,900)	1,811 (0 – 92,200)	2,644 (0 – 160,400)	6,812 (0 – 200,300)
Spouse/partner's education, some graduate school, N (%)	1000 (24.2)	1315 (30.3)	1344 (31.37)	1518 (33.7)	1377 (34.9)
Smoking during index pregnancy, N (%)	300 (6.9)	293 (6.4)	272 (6.0)	374 (7.8)	331 (7.9)
Family income, $50,000-74,000, N (%)	1085 (30.8)	1051 (29.0)	906 (25.2)	914 (24.0)	737 (22.5)

†Numbers in the range have been rounded to protect participant anonymity.

Supplemental Material, Table S3: Odds ratio for quintile 5 vs quintile 1 of autism spectrum disorder with overall metals measure, sensitivity analyses, children of the Nurses' Health Study II†

	OR (95% CI)	P-value trend
Base model	1.62 (1.08, 2.44)	0.01
+ Smoking during index pregnancy	1.68 (1.05, 2.67)	0.05
+ Income in 2001	1.65 (1.04, 2.62)	0.02
+ Spouse/partner's education	1.57 (1.02, 2.40)	0.03
1990 HAP year only	1.42 (0.85, 2.38)	0.19
1996 HAP year only	1.88 (0.84, 4.18)	0.02
Largest 6 states only, adjusted for state	2.19 (1.26, 3.83)	0.02
Rural quartile only	1.12 (0.37, 3.38)	0.89
Urban quartile only	10.07 (1.28, 79.15)	0.03
+ Population density	1.55 (1.02, 2.37)	0.03

†All models adjusted for sex, maternal age at birth, year of birth, Census tract % college educated, Census tract median income, and adjusted for or restricted by HAP model year.

Supplemental Material, Table S4: Odds ratio [highest versus lowest quintile (95% confidence interval)] of autism spectrum disorder in multiple-pollutant and single-pollutant models, children of the Nurses' Health Study II, born 1987-2002†

	Models with one pollutant (N cases= 105, N controls=8663)	Model with multiple pollutants, excluding diesel (N cases=105, N controls =8663)	Model with multiple pollutants, excluding diesel, **boys only** (N cases=98, N controls=4363)
Cadmium	1.5 (1.0, 2.1)	0.9 (0.5, 1.6)	1.0 (0.6, 1.8)
Lead	1.6 (1.1, 2.3)	1.4 (0.8, 2.5)	1.6 (0.9, 2.9)
Manganese	1.5 (1.1, 2.2)	1.0 (0.6, 1.7)	0.7 (0.4, 1.4)
Nickel	1.7 (1.1, 2.5)	1.3 (0.8, 2.2)	1.5 (0.8, 2.7)
Diesel	2.0 (1.0, 4.0)	--	--
Methylene Chloride	1.5 (1.0, 2.1)	1.1 (0.7, 1.8)	1.2 (0.7, 2.0)

†Models adjusted for maternal age at birth, year of birth, maternal parents' education, Census tract median income, Census tract % college educated, HAP model year and sex. Diesel was excluded from multiple pollutant models presented here because diesel data were available for only 4,843 respondents.

Supplemental Material, Table S5: Odds ratio of autism in child, highest pollutant concentration quintile versus lowest quintile exposure, children of the Nurses' Health Study II (n=22,442), listed by *P*-value.†

	**Odds ratio (95% CI)**	**P-value**
Beryllium compounds	1.77 (1.22, 2.57)	0.003
Ethylene dichloride	2.14 (1.26, 3.62)	0.005
Acetonitrile	1.73 (1.16, 2.59)	0.007
1,2,4-Trichlorobenzene	1.58 (1.12, 2.21)	0.008‡
**Nickel compounds***	**1.65 (1.10, 2.47)**	**0.01**
Propylene oxide	1.81 (1.11, 2.96)	0.02
1,3-Butadiene	1.57 (1.08, 2.28)	0.02
Hydrazine	1.47 (1.07, 2.03)	0.02‡
**Lead compounds***	**1.57 (1.09, 2.27)**	**0.02**
**Manganese compounds***	**1.54 (1.07, 2.23)**	**0.02**
Toluene	1.56 (1.06, 2.30)	0.02
Tetrachloroethylene (Perchloroethylene)	1.60 (1.07, 2.41)	0.02
Phthalic anhydride	1.75 (1.07, 2.85)	0.03
Cyanide compounds	1.64 (1.06, 2.53)	0.03
Ethylene oxide	1.50 (1.03, 2.20)	0.03
Vinyl acetate	1.71 (1.06, 2.75)	0.03
Dimethyl carbamoyl chloride	2.63 (1.07, 6.48)	0.04††
Chlordane	0.17 (0.03, 0.92)	0.04
**Diesel particulate matter***	**2.01 (1.02, 3.97)**	**0.04**
Hexane	1.50 (1.03, 2.18)	0.04
Methylene_bis (2-chloroaniline)	1.51 (1.02, 2.24)	0.04††
Polycyclic Organic Matter	1.61 (1.02, 2.52)	0.04
Benzene	1.48 (1.01, 2.17)	0.047
**Cadmium compounds***	**1.46 (1.00, 2.13)**	**0.048**
Maleic anhydride	1.60 (1.00, 2.56)	0.048
Ethyl acrylate	1.63 (1.00, 2.67)	0.052
**Methylene chloride***	**1.46 (0.99, 2.15)**	**0.054**
Acrylonitrile	1.49 (0.98, 2.26)	0.06
Ethyl benzene	1.43 (0.98, 2.09)	0.06
1,2-Dibromo-3-chloropropane	2.36 (0.94, 5.95)	0.07
1,3-Dichloropropene	1.44 (0.97, 2.14)	0.07
Ethyl chloride	1.58 (0.97, 2.58)	0.07
Methylene diphenyl diisocyanate	1.48 (0.97, 2.25)	0.07
Acetaldehyde	1.43 (0.96, 2.15)	0.08
Acrolein	1.43 (0.96, 2.12)	0.08
Asbestos	2.20 (0.92, 5.28)	0.08‡
Formaldehyde	1.43 (0.96, 2.12)	0.08
Phosphine	2.87 (0.87, 9.43)	0.08

	**Odds ratio (95% CI)**	**P-value**
2,2,4-Trimethylpentane	1.47 (0.95, 2.27)	0.09
Phosgene	1.62 (0.93, 2.82)	0.09
**Styrene***	**1.40 (0.95, 2.07)**	**0.09**
Titanium tetrachloride	3.90 (0.82,18.64)	0.09
Trifluralin	0.65 (0.39, 1.07)	0.09[††]
Chloroprene	1.54 (0.92, 2.57)	0.10
**Antimony compounds***	**1.43 (0.92, 2.21)**	**0.11**
Cresols_Cresylic acid (isomers and mixture)	1.45 (0.92, 2.27)	0.11
**Mercury compounds***	**1.37 (0.93, 2.00)**	**0.11**
Carbon tetrachloride	1.66 (0.89, 3.08)	0.11
2,4-Toluene diisocyanate	1.39 (0.92, 2.09)	0.12
**Chromium compounds ***	**1.36 (0.93, 1.98)**	**0.12**
Hexachlorobutadiene	2.36 (0.79, 7.03)	0.12
Vinylidene chloride	1.39 (0.91, 2.11)	0.12
Carbonyl sulfide	1.55 (0.88, 2.76)	0.13
Xylenes (isomers and mixtures)	1.34 (0.92, 1.94)	0.13
4,4'-Methylene bis(2-chloroaniline)	0.34 (0.08, 1.44)	0.14[††]
Benzyl chloride	1.28 (0.92, 1.79)	0.14‡
POM Group 2: no URE data	0.20 (0.02, 1.71)	0.14
Aniline	1.34 (0.90, 2.00)	0.15
4-Nitrophenol	0.62 (0.31, 1.26)	0.18‡
**Arsenic compounds** (inorganic, may include arsine)*	**1.32 (0.88, 1.96)**	**0.18**
Chromium III	3.04 (0.60, 15.42)	0.18
Methoxychlor	0.69 (0.39, 1.21)	0.19[††]
**Trichloroethylene**	**1.32 (0.86, 2.02)**	**0.21**
2,4-D, salts and esters	0.77 (0.50, 1.17)	0.22[††]
Polychlorinated biphenyls (PCBs)	1.30 (0.85, 1.96)	0.22
Bis(2-ethylhexyl)phthalate	1.34 (0.83, 2.15)	0.23
Chloroacetic acid	0.79 (0.53, 1.17)	0.23‡
Coke Oven Emissions	0.74 (0.45, 1.22)	0.23‡
Hexachlorobenzene	1.39 (0.81, 2.37)	0.23‡
Methyl tert butyl ether	1.23 (0.87, 1.76)	0.24
4,4'-Methylenedianiline	1.23 (0.87, 1.74)	0.25‡
Propoxur	1.57 (0.73, 3.39)	0.25[††]
3,3-Dichlorobenzidene	1.24 (0.85, 1.82)	0.26[††]
Carbaryl	0.77 (0.49, 1.22)	0.26[††]
Epichlorohydrin	1.21 (0.87, 1.69)	0.26‡
Propionaldehyde	1.24 (0.85, 1.83)	0.26
Carbon disulfide	1.26 (0.83, 1.93)	0.28
Methyl chloroform	1.29 (0.81, 2.04)	0.28
Methyl methacrylate	1.30 (0.81, 2.07)	0.28

	Odds ratio (95% CI)	P-value
Dibenzofurans	2.06 (0.54, 7.77)	0.29
Methanol	1.27 (0.82, 1.97)	0.29
PCDD/PCDFs	1.28 (0.81, 2.02)	0.30
Chloromethyl methyl ether	1.34 (0.76, 2.38)	0.31††
Diazomethane	3.05 (0.36,25.74)	0.31††
Hexamethylene-1,6-diisocyanate	1.49 (0.66, 3.35)	0.33‡
Dichlorvos	0.74 (0.39, 1.40)	0.35††
7-PAH	1.37 (0.69, 2.73)	0.36
Propylene dichloride	1.24 (0.78, 1.97)	0.36
2,4,6-Trichlorophenol	0.64 (0.23, 1.79)	0.39††
Heptachlor	0.71 (0.31, 1.61)	0.41††
Quinone	1.26 (0.73, 2.17)	0.41††
Glycol ethers	1.22 (0.77, 1.92)	0.42
Chlorobenzene	1.21 (0.76, 1.93)	0.43
POM Group 8: Unspeciated 7-PAH	0.63 (0.20, 2.01)	0.43
Benzidine	0.57 (0.13, 2.43)	0.44††
Dichloroethyl ether	0.81 (0.47, 1.39)	0.45††
N,N-Diethyl aniline	1.59 (0.47, 5.35)	0.45
POM Group 1: Unspeciated	1.76 (0.41, 7.57)	0.45
Ethyl carbamate	1.13 (0.82, 1.57)	0.46††
Hydrochloric acid	1.18 (0.76, 1.82)	0.46
Pentachloronitrobenzene	2.65 (0.17, 40.64)	0.48††
2-Nitropropane	1.16 (0.76, 1.77)	0.49††
4-Nitrobiphenyl	1.68 (0.38, 7.33)	0.49‡
Acetophenone	1.19 (0.73, 1.93)	0.49††
Acrylic acid	1.15 (0.78, 1.69)	0.49‡
Arsine	1.55 (0.45, 5.33)	0.49††
**Vinyl chloride***	**1.17 (0.75, 1.81)**	**0.49**
2,4-Toluene diamine	1.16 (0.76, 1.77)	0.50††
Ethylene dibromide	2.02 (0.27, 15.20)	0.50
Ethylene thiourea	1.13 (0.78, 1.62)	0.51††
2-Acetylaminofluorene	0.52 (0.07, 3.89)	0.52††
N,N-diethyl/dimethylaniline	1.12 (0.79, 1.58)	0.53
p-Dichlorobenzene	1.16 (0.73, 1.86)	0.53
Acetamide	1.77 (0.28, 11.28)	0.54††
Dimethyl sulfate	1.11 (0.79, 1.56)	0.54‡
Styrene oxide	1.18 (0.69, 2.02)	0.54††
Cobalt compounds	1.13 (0.74, 1.72)	0.56
Dibutylphthalate	1.14 (0.74, 1.78)	0.57
Diethanolamine	1.12 (0.75, 1.67)	0.57
Acrylamide	1.10 (0.78, 1.56)	0.58‡

	Odds ratio (95% CI)	P-value
Methyl hydrazine	1.10 (0.78, 1.55)	0.58‡
1,4-Dioxane	1.10 (0.77, 1.57)	0.60‡
Bromoform	1.15 (0.68, 1.97)	0.60
Hexachlorocyclopentadiene	0.88 (0.55, 1.43)	0.61††
Hydrofluoric acid	1.11 (0.75, 1.64)	0.61
4,6-Dinitro-o-cresol, and salts	0.86 (0.47, 1.56)	0.62‡
1,2-Propylenimine	1.11 (0.72, 1.70)	0.64††
Hydroquinone	1.08 (0.77, 1.52)	0.64‡
N-Nitrosomorpholine	1.64 (0.21, 12.77)	0.64††
o-Toluidine	1.09 (0.76, 1.58)	0.64‡
MEK_total	1.11 (0.69, 1.78)	0.66
Methyl isobutyl ketone	1.10 (0.71, 1.69)	0.67
1,1-Dimethyl hydrazine	1.20 (0.50, 2.91)	0.68††
Pentachlorophenol	1.08 (0.75, 1.55)	0.68‡
Bis(chloromethyl)ether	0.89 (0.51, 1.56)	0.69††
POM Group 3: 5.0E-2<URE<=5.0E-1	1.37 (0.29, 6.35)	0.69
Triethylamine	1.27 (0.41, 3.95)	0.69
Chromium VI	0.76 (0.17, 3.46)	0.72
Cumene	1.08 (0.68, 1.70)	0.74
Toxaphene	0.70 (0.08, 5.87)	0.74††
Selenium Compounds	0.93 (0.60, 1.44)	0.75
Phenol	1.08 (0.66, 1.74)	0.76
POM Group 7: 5.0E-6<URE<=5.0E-5	1.24 (0.32, 4.75)	0.76
2,4-Dinitrophenol	0.94 (0.63, 1.41)	0.77††
Benzotrichloride	1.07 (0.67, 1.72)	0.78††
Nitrosodimethylamine	0.81 (0.19, 3.52)	0.78††
1,1,2,2-Tetrachloroethane	1.07 (0.63, 1.82)	0.79‡
Ethylene glycol	1.07 (0.66, 1.73)	0.79
Methyl isocyanate	0.91 (0.48, 1.76)	0.79††
o-Anisidine	0.89 (0.38, 2.07)	0.79††
POM Group 6: 5.0E-5<URE<=5.0E-4	1.20 (0.31, 4.62)	0.79
1,2-Epoxybutane	1.05 (0.73, 1.51)	0.80
2-Chloroacetophenone	0.89 (0.35, 2.24)	0.80‡
2,4,5-Trichlorophenol	1.16 (0.34, 3.95)	0.81††
Chlorine	0.87 (0.28, 2.67)	0.81
Chloroform	1.04 (0.73, 1.49)	0.82
3,3-Dimethoxybenzidine	1.06 (0.60, 1.88)	0.84††
4-Aminobiphenyl	1.24 (0.16, 9.61)	0.84‡
Hexachloroethane	0.81 (0.11, 6.19)	0.84
Ethylidene dichloride	0.89 (0.29, 2.80)	0.85††
Isophorone	1.13 (0.34, 3.72)	0.85

	Odds ratio (95% CI)	P-value
Methyl chloride	1.05 (0.64, 1.72)	0.86
Methyl iodide	1.13 (0.31, 4.05)	0.86
1,1,2-Trichloroethane	1.04 (0.63, 1.72)	0.87
Nitrobenzene	1.03 (0.72, 1.48)	0.87‡
POM Group 5: 5.0E-4<URE<=5.0E-3	0.89 (0.22, 3.66)	0.87
2,4-Dinitrotoluene	1.03 (0.64, 1.66)	0.89[††]
Biphenyl	0.97 (0.63, 1.50)	0.89
**Quinoline***	**1.02 (0.73, 1.43)**	**0.89‡**
p-Phenylenediamine	1.03 (0.70, 1.51)	0.90[††]
Dimethyl phthalate	1.02 (0.72, 1.44)	0.92‡
Methyl ethyl ketone	0.94 (0.27, 3.26)	0.92
Dimethyl formamide	0.98 (0.65, 1.48)	0.93‡
Catechol	1.03 (0.50, 2.10)	0.94[††]
Fine mineral fibers	0.93 (0.12, 7.21)	0.94[††]
Allyl chloride	1.01 (0.70, 1.44)	0.97‡
Lindane (all isomers)	0	0.97
Methyl bromide	1.02 (0.21, 4.97)	0.98
N-Nitroso-N-methylurea	0	0.98[††]
Chloramben	0	0.99[††]
Hexamethylphosphoramide	0	0.99[††]
1,2-Diphenylhydrazine	Not estimable	
1,3-Propane sultone	Not estimable	
3,3-Dimethyl benzidine	Not estimable	
beta-Propiolactone	Not estimable	
Calcium cyanamide	Not estimable	
Captan	Not estimable	
Chlorobenzilate	Not estimable	
DDE (1,1-Dichloro-2,2-Bis(P-Chlorophenyl)	Not estimable	
Ethylene	Not estimable	
Diethyl sulfate	Not estimable	
Ethylene imine	Not estimable	
N,N-Dimethyl aniline	Not estimable	
Napthalene	Not estimable	
Parathion	Not estimable	
p-Dimethylaminoazobenzene	Not estimable	
Phosphorous	Not estimable	
POM Group 4: 5.0E-3<URE<=5.0E-2	Not estimable	
Vinyl bromide	Not estimable	

†Models adjusted for maternal age at birth, year of birth, maternal parents' education, Census tract median income, Census tract % college educated, HAP model year and sex.
*Pollutants selected *a priori* in bold font.

Pollutants listed above the double line are significant at $p<0.05$.
Note: Of pollutants selected *a priori*, antimony was not available in the 1996 model year, chromium was not available in the 1999 model year, and diesel was not available in the 1990 model year. The other *a priori* pollutants were available in each year. Of the remaining 198 pollutants, only 27 were available for the 1996 model year (relevant to 15% of our sample). For the 1990 model year, 46 of these 198 pollutants were not available; for the 1999 model year, 8 were not available; and for the 2002 model year, 16 were not available.
‡The distribution of this chemical did not permit formation of quintiles. We therefore compared the highest quartile to the lowest quartile.
††The distribution of this chemical did not permit formation of quintiles or quartiles. We therefore compared the highest tertile to the lowest tertile.

All *EHP* content is accessible to individuals with disabilities. A fully accessible (Section 508–compliant) HTML version of this article is available at http://dx.doi.org/10.1289/ehp.122-A152.

Erratum: "Perinatal Air Pollutant Exposures and Autism Spectrum Disorder in the Children of Nurses' Health Study II Participants"

Roberts et al. have reported errors in their article "Perinatal Air Pollutant Exposures and Autism Spectrum Disorder in the Children of Nurses' Health Study II Participants" [Environ Health Perspect 121:978–984 (2013); http://dx.doi.org/10.1289/ehp.1206187]. In hazardous air pollutant (HAP) data used in their paper, U.S.-wide background concentrations were not added to the concentration estimates for 13 HAPs [benzene, carbon tetrachloride, chloroform, ethylene dibromide, ethylene dichloride, formaldehyde, hexachlorobenzene, mercury, methylene chloride, polychlorinated biphenyls, tetrachloroethylene (perchloroethylene), trichloroethylene, and xylene] in the 1996 HAP model year. As a result, effect estimates reported for quintiles of these 13 HAPs formed using all 4 HAP years were in fact effect estimates for somewhat different percentile comparisons; for example, results shown for the comparison of the highest versus lowest quintile of mercury were actually closer to the highest quartile versus the lowest 5%. Results for mercury, methylene chloride, and tetrachloroethylene were incorrect in Figure 1 and Table 2, and several values were incorrect in Figure 2. Because mercury was a constituent (one of eight) of the pooled and overall metals measures, corrected results related to pooled metals and overall metals quintiles in Table 2 are slightly different, as are results in Supplemental Material. In addition, the median—rather than the mean—tract population density was inadvertently entered for those without autism spectrum disorder in Table 1; the correct mean value is 2,578 persons/mi^2.

The changes largely do not affect the conclusions of the study, except that the results for mercury and methylene chloride for both sexes combined did not quite reach statistical significance at $p < 0.05$. In addition, in multipollutant models, the odds ratio for lead became the strongest, rather than mercury and methylene chloride, as the authors reported in the original version of the paper.

The article, including tables and figures, as well as the Supplemental Material have been corrected online.

The authors regret the errors.
